# Supplementary material for: Polyphenols in Jabuticaba (Plinia spp.) Peel Flours: Extraction and Comparative Evaluation of FTIR and HPLC for Quantification of Individual Compounds
Source: Foods. 2023 Apr 1;12(7):1488. doi: 10.3390/foods12071488 (PMC10094258; doi:10.3390/foods12071488)
Supplement: Supplementary file 1 [file foods-12-01488-s001.zip › foods-2251499-supplementary.pdf]

Supplementary Data

Table S1 – Jabuticaba samples used in this study

Sample	Origin information		Mass (kg)
	City - neighborhood	District	
**S1**	Belo Horizonte	Belo Horizonte	1.56
**S2**	Belo Horizonte	Belo Horizonte	4.23
**S3**	Entre Rios de Minas – Comunidade Rural Camapuã de Cima	Conselheiro Lafaiete	3.20
**S4**	Mateus Leme – Bairro Santa Bárbara	Belo Horizonte	2.70
**S5**	Mateus Leme – Bairro Centro	Belo Horizonte	3.55
**S6**	Florestal	Pará de Minas	1.86
**S7**	São José da Lapa	Belo Horizonte	3.16
**S8**	Entre Rios de Minas – Comunidade Rural Água Limpa	Conselheiro Lafaiete	2.10
**S9**	Entre Rios de Minas – Bairro Centro	Conselheiro Lafaiete	2.13
**S10**	Mariana	Ouro Preto	2.14
**S11**	Luz	Bom Despacho	2.40
**S12**	Mateus Leme – Comunidade Rural Sítio Novo	Belo Horizonte	2.41
**S13**	Rio Piracicaba	Itabira	1.36
**S14**	São Gonçalo do Rio Abaixo	Itabira	1.25
**S15**	Bom Jesus do Amparo	Itabira	1.38
**S16**	Entre Rios de Minas – Comunidade Rural Água Limpa	Conselheiro Lafaiete	2.59
**S17**	Desterro de Entre Rios	Conselheiro Lafaiete	2.64
**S18**	Lagoa Dourada	São João del Rei	2.44
**S19**	Sabará – Bairro Paciência	Belo Horizonte	1.38
**S20**	Entre Rios de Minas – Bairro Castro	Conselheiro Lafaiete	2.19
**S21**	Resende Costa	São João del Rei	1.04
**S22**	Jeceaba	Itaguara	1.78
**S23**	Sabará	Belo Horizonte	1.67
**S24**	Itabirito	Ouro Preto	1.48
**S25**	Itabira	Itabira	1.69
**S26**	Nova Era	Itabira	1.66
**S27**	Mateus Leme - Bairro Santa Bárbara	Belo Horizonte	1.22
**S28**	Inimutaba	Curvelo	1.14
